# Mr. G. Q. Roberts's Interesting Lecture

**Published:** 1920-06-05

**Authors:** 


### HISTORIC HOSPITALS.

### Mr. G. Q. ROBERTS'S INTERESTING LECTURE.

Some months ago Mr. G. Q. Roberts, C.B.E., M.A., Secretary of St. Thomas's Hospital, gave a lecture on "Old London and the London Hospitals" to a gathering of medical men in the city. It was so much appreciated that Mr. Roberts repeated it, by request, for the benefit of members and workers of the League of Mercy, a large number of whom gathered at St. Thomas's Hospital on May 28. Sir William Collins, who presided, remarked that it was good to know that Londoners, though tardily, were awaking to the historical interest of the city. The criticism with which the report of the Commission regarding City churches was received, showed that Londoners did take a pride in their venerable institutions. Sir William recalled a saying of Mr. Passmore Edwards: "Hospitals are as sacred as churches, and nurses are entitled to as much, if not more, consideration than parsons." From what they read in the Press they might be inclined to think the hospitals' necessity was the State's opportunity, and that voluntary hospitals, like some of the City churches, might be doomed. A strong effort would, however, be made to maintain the voluntary system in London.

Mr. Roberts, in spite of a severe cold, gave a most graphic and interesting lecture, which was illustrated by a series of excellent lantern slides. The first of the latter was quite up to date, showing St. Thomas's Hospital from the air. The lecturer, however, quickly took his audience back to the thirteenth century, when the great religious houses already specialised in hospital work, and explained how, when Henry VIII. closed all religious institutions, London suffered intensely. In response to urgent petitions the King restored St. Bartholomew's and St. Thomas's, also Bridewell, for the correction of malefactors, Christ's Hospital for the education of neglected and fatherless children, and St. Mary's of Bethlehem for the cure of the insane. The management of these hospitals was put into the hands of the Corporation of London, and it was interesting to note that the citizens of those days deliberately set to work to raise funds, every householder being systematically called upon, and sermons—often written for and supplied to the preachers—being delivered in every church. These were the only hospitals at that time, except those for lepers.

Some exceedingly interesting old maps and drawings—one by a clever patient in the modern Bethlehem hospital—were thrown on the screen as Mr. Roberts traced the history of St. Thomas's and described the famous building which formerly stood in Southwark, and which was sold to the South-Eastern Railway Company in 1862. The great hospitals were originally situated in rural surroundings, and the London Hospital was at one time described as being "on the quiet road leading from the City of London to the pleasant village of Mile End"! Mr. Roberts had much that was interesting to say about the great improvements made at the London Hospital since the 'eighties, especially in the matter of sanitation. He mentioned the fact that Miss Florence Nightingale did a great work in connection with St. Thomas's in Southwark. Nothing could have been more effective in the way of contrast than two pictures thrown on the screen, one showing the conditions under which the "Lady of the Lamp" worked at Scutari, the other showing an up-to-date hospital ward full of wounded soldiers. Photographs were shown of the old-fashioned curtained beds that were formerly used, and of wards crowded with dust-collectors, in contrast to the light, airy wards of the modern hospital.

In concluding a fascinating lecture, packed with both entertainment and instruction, Mr. Roberts said some people might think the Government should take over the hospitals, but the voluntary system had existed all through the centuries, and those who had seen something of the work of institutions under Government control could only urge that it was in the best interests of the community to keep the voluntary principle alive. A vote of thanks was accorded with acclamation to the lecturer. Sir William Collins remarked that Mr. Roberts had told how an American visitor to the House of Commons once mistook St. Thomas's noble pile for "the houses of the aristocracy." That reminded him that when St. George's, St. Bart's, Guy's, and St. Thomas's were the four chief medical schools in London they were commonly classified as follows: "The gentlemen of St. George's, the students of Bart's, and the cads of the Boro'." When St. Thomas's moved West, it evidently joined the aristocracy! Sir William concluded the proceedings by heartily commending the good work of the League of Mercy to any who were not already members of that splendid organisation.

